# Could life story work support relational autonomy in advance care planning? Stories from the EARLI project

**DOI:** 10.1111/ajag.70042

**Published:** 2025-05-02

**Authors:** Ava Karusoo‐Musumeci, Ling Yeoh, Caroline Edwards, Rebecca Walton, Melanie Crabtree, Michelle M. Hilgeman, Craig Sinclair

**Affiliations:** ^1^ Neuroscience Research Australia Sydney New South Wales Australia; ^2^ University of New South Wales Sydney New South Wales Australia; ^3^ Brightwater Group Perth Western Australia Australia; ^4^ The University of Alabama Tuscaloosa Alabama USA; ^5^ Tuscaloosa Veterans Affairs Medical Center Tuscaloosa Alabama USA

**Keywords:** advance care planning, aged, relational autonomy

## Abstract

**Objectives:**

Advance care planning has evolved from a narrow focus on advance directives completion towards a greater emphasis on ongoing conversations and value clarification. This evolution aligns with a relational perspective on autonomy in a social context. However, limited research explores how relational autonomy might be operationalised in practice. Life story work is a novel approach that may serve to operationalise relational autonomy in advance care planning.

**Methods:**

This paper presents three vignettes from the Enhanced Advance Care Planning and Life Review Longitudinal Intervention (EARLI) project, an arts‐based intervention that uses life story work to support advance care planning among older adults living in the community.

**Results:**

The vignettes illustrated how life story work facilitated discussions about values and preferences, enhanced relational autonomy and influenced participants' engagement with advance care planning across different stages.

**Conclusions:**

Integrating life story work into advance care planning provides a practical approach to fostering relational autonomy. This method offers insight into the ways personal narratives can shape decision‐making and communication within the advance care planning process.

**Practice impact**

This paper explored a novel approach to advance care planning using life story work. Vignettes illustrated how this process can operationalise a relational autonomy approach to advance care planning with older adults in community settings.

## 1 INTRODUCTION

Family photographs were presented at the end of Mrs. Chen's short life story book, while each chapter featured photographs of her prized flowers. The front cover read *My life story and wishes for my future care* and was adorned with a flourish of purple and white orchids. A pot of pink blossoms decorated *My Spiritual Faith* and *My health and wellbeing*. The chapter *My end‐of‐life wishes* featured a pure white orchid disappearing into a clean white background. Mrs. Chen was a participant in the Enhanced Advance Care Planning and Life Review Longitudinal Intervention (EARLI) project,^1^ a randomised controlled trial exploring a combined life story work (LSW) and advance care planning (ACP) program among older adults living in the community. Over four visits across roughly 2 months, Mrs. Chen and a facilitator worked together on her LSW, using the storytelling process to enable discussion about future care.

This paper shares the experiences of three participants, Mrs. Chen, Mrs. Dixon and Mrs. Tripoli (pseudonyms), in this program. Participants provided consent for the recording of study sessions and use of their life stories. These ‘stories about stories’ explored how this arts‐based intervention might assist in operationalising relational autonomy across the ACP continuum.

The EARLI project was motivated by low ACP uptake among older adults in community settings, with facilitated ACP interventions having only modest impact.^2^, ^3^ With an ageing population, ACP has become increasingly vital. Longer lifespans often bring complex health challenges, including chronic illness, physical decline, caregiver stress and greater reliance on health‐care services.^4^ Advance care planning increases agreement between the end‐of‐life care patients receive and their stated preferences,^5^ as well as quality of dying.^6^ However, uptake of ACP globally remains low.^7^

One explanation for lower uptake is that the conceptualisation of autonomy in ACP does not suit the actual practice of end‐of‐life decision‐making.^8^ Advance care planning was initially focused on documenting end‐of‐life treatment preferences; however, it has since evolved into a continuum of care planning that begins when a person considers their future care and continues throughout their care.^9^ This process‐oriented view shifts ACP's objectives from recording advance treatment decisions to educating and empowering people to engage in end‐of‐life decision‐making.^10^

### 1.1 Relationality across the ACP continuum

It has been proposed that individual autonomy serves as an underlying philosophy for much of ACP globally.^11^, ^12^ Individual autonomy is based on principles of informed consent and implies that decisions are made by an independent person based on rational self‐interest without the undue influence of others.^8^ This approach to autonomy in health care improved patient outcomes by protecting against paternalism and futile treatments, promoting patient rights such as privacy, confidentiality and self‐determination, and enhancing empowerment in end‐of‐life care.^13^ However, an individual approach to autonomy tends to overlook how real‐world decisions are influenced by numerous interpersonal and contextual factors, including culture, family, social connections and past experiences.^14^ Decisions about end‐of‐life are also frequently made with emotion rather than rationality.^11^ Additionally, the individual emphasis on autonomy can be inappropriate within some cultural settings.^15^, ^16^ Due to these factors, researchers are increasingly redefining autonomy in ACP as relational.

A relational approach to autonomy considers a person's whole context when making decisions and may support people from collectivist cultures to participate in ACP. Relational autonomy is a multi‐source concept with roots in several ethical and political‐philosophical approaches.^13^ These approaches consider that decisions and decision‐making processes occur and develop within a social context and that this context as well as a person's historical roots impacts their expression of autonomy. A multitude of aspects may define a person and their experience of illness, including their social location, identities, roles and relationships.^8^ A relational approach to autonomy is recommended by many working within collectivist cultures, where family‐based decision‐making is favoured.^15^, ^16^, ^17^

Some studies have found that while health practitioners believe in supporting individual autonomy, in practice they base end‐of‐life decisions on relationships between physicians, patients and family.^18^, ^19^, ^20^ This may be due to differing perspectives on the intention of ACP, with patients viewing the process as a means to ensure their final wishes are enacted, and doctors viewing ACP documents as an unreliable indicator of the person's wishes that potentially inhibits quality care.^20^ This perspective, that ‘autonomy and end‐of‐life care is relational’,^18^ suggests that the ACP process might be more effective if it can also be consistent with a relational perspective on autonomy.^18^, ^20^

### 1.2 Life story in ACP

Taking a relational approach to autonomy across the continuum of ACP shows theoretical promise, yet little has been done to explore how this may be operationalised in practice. The creative practice of LSW may serve as an effective tool to fill this gap. Life story work constitutes one of the oldest forms of art in human history.^21^ While LSW can be used as a therapeutic tool, such as in reminiscence therapy,^22^ the EARLI approach to the practice is non‐therapeutic. Life story work in the health environment is a creative process of sharing and recording life stories.^23^ The LSW process has been shown to benefit the person, their family and health‐care staff through preserving identity, improving relationships, enhancing person‐centred care, promoting communication and increasing quality of life.^23^, ^24^, ^25^, ^26^

Several of these benefits also support the integration of relational autonomy into ACP. Thoresen and Lillemoen^27^ ^, p. 11^ note that a patient's family plays an important role in ACP as ‘they know about the patient's life story, and they [are] also able to make meaningful connections between the patients’ former life and the present situation’. Understanding the whole person, their values and goals when discussing ACP is potentially beneficial to health‐care practitioners^24^ and may further support relational approaches to ACP through preserving identity, improving relationships, identifying appropriate substitute decision‐makers and ensuring documentation is sensitive to a person's social and relational contexts.

Few studies have explored the integration of LSW into ACP discussions. The Preserving Identity and Planning for Advance Care (PIPAC) study^28^ used LSW to facilitate ACP with people with early‐stage dementia. Sessions involved the creation of a life story product, transitioning discussion from ‘what it has meant to live well in the past’ to ‘what it will mean to live well in the future’. Further studies have explored the use of life story work in collaboration with other ACP discussion strategies to promote culturally adapted ACP for advanced cancer patients.^29^ Given the promise of this emerging practice, we describe three vignettes that illustrate how the EARLI project integrates LSW and ACP, supporting a relational approach to autonomy in practice.

## 2 METHODS

The EARLI project was an open‐label, cross‐over, cluster‐randomised controlled trial conducted across 12 aged care organisations in New South Wales and Western Australia.^1^ The trial included participants aged 65 years and older who received home care services and were able to provide informed consent at the time of recruitment. Participants were recruited from November 2022 to March 2025.

The vignettes presented in this paper were derived from the experiences of three participants in the intervention group. The intervention involved four visits between the participant and facilitator of approximately 1 h in length, focusing on the participant's life story as well as their wishes for future care (Appendix S1). Facilitators had backgrounds in speech pathology, occupational therapy and nursing, and received training totalling 22 h in project methods, trauma‐informed practice, LSW and ACP facilitation. The LSW was developed progressively in collaboration between the participant and facilitator. Participants could choose the content and format of their LSW and ACP documents. Full details on the intervention have been published.^1^

Vignettes were selected for inclusion in this article as they represented the diversity of participants and their approaches to both LSW and ACP. The participants represent the three language groups accepted in the project: English, Chinese (Mandarin or Cantonese) and Italian. Participant stories were synthesised through review of audio‐recorded study visits, facilitator notes, as well as LSW and ACP documents created in the project. Pseudonyms reflecting the participants' cultural heritages have been used to maintain confidentiality.

Ethics approval was received from the UNSW Human Research Ethics Committee (HC220271).

## 3 RESULTS

### 3.1 Mrs. Dixon

Mrs. Dixon, aged 90, used her time in the EARLI project to complete a life story on the theme of ‘motherhood’, exploring personal stories of childrearing. Through the storytelling process, the facilitator learned about her deep Catholic faith, the importance of family and how these shaped her worldview. Mrs. Dixon expressed anxiety about how ACP might align with her religious beliefs, particularly the notion of control over life and death. She was encouraged to seek guidance from her priest for reassurance about her religious concerns. She also sought advice from her doctor to explore the medical meaning of ‘life support’ in greater detail.

Mrs. Dixon was uneasy about making advance treatment decisions, as she felt her circumstances might change over time. She declined a formal directive and instead sent an informal ‘plan’ by email to her children. This document would guide them in making decisions on her behalf, without strictly dictating the course of action. In this email, she explained the background of her decision, and the advice she had received from both her priest and her doctor. She also discussed the advice of the facilitator and explained her reasoning for choosing a plan over an official directive. Consistent with her life story theme on motherhood, she nominated two of her six children to be her substitute decision‐makers, as they shared her Catholic faith. She also highlighted the importance of time, suggesting she would like to be kept alive for significant family events such as weddings, even if it meant family would need to visit her in hospital.

### 3.2 Mrs. Chen

Mrs. Chen, aged 91, completed her sessions in Mandarin with a facilitator fluent in the language. Her experience highlighted the merging of the LSW and ACP processes throughout the EARLI project. Rather than LSW acting as a preamble to ACP, the two conversations combined, often occurring simultaneously with ACP discussions inviting anecdotes and reflections on past experiences. The self‐titled chapters of Mrs. Chen's LSW crossed between both LSW and ACP. In *Important people in my life*, Mrs. Chen shared her gratitude to her sons for their care for her husband at his end‐of‐life, when he was admitted to residential aged care. Later, she wrote a section titled *My health and wellbeing* where she listed her ailments, followed by a request to stay at home as long as possible. She again reflected on her faith in her sons, noting she was confident they would take good care of her ‘when the time comes’.

Mrs. Chen wrote her LSW by hand, which was later typed in English and Chinese by the study facilitator. It may be that penning her life story helped her to make end‐of‐life decisions based on her values, principles and lived experiences. Initially, she declined to include photographs; however, when presented with a draft of the LSW containing stock photographs, she began to take ownership of the process, directing the facilitator to photograph her flowers and determining where they would be placed in the story. A similar transition took place regarding her engagement with ACP. At the beginning of the project, she did not understand ACP. Later, she began to engage but declined to involve her doctor. By the end of the project, she expressed a desire to discuss ACP with her doctor and ultimately completed an advance care directive.

### 3.3 Mrs. Tripoli

Sessions with Mrs. Tripoli, aged 79 and born in Italy, took place in two distinct halves. The first half of each session followed the standard EARLI project procedure (Appendix S1).^1^ The second half of each session involved chatting over espresso and biscotti. This part of each visit was relaxed and often continued the conversation about ACP. Mrs. Tripoli's sessions always included various other people. Her husband sat in on all sessions, and at different times, her daughters would join or her grandson. Sessions with Mrs. Tripoli were exuberant family affairs, far removed from a clinical process of health‐care documentation.

Mrs. Tripoli had already nominated her husband as a substitute decision‐maker, with her daughters as alternatives. As well as a life story book, she completed a formal directive at the conclusion of the study; however, she was not confident in making advance treatment decisions regarding resuscitation and life support. She declined to fill this section in and opted to only complete part of the form, which allowed her to share her values, indicating conditions she would find bearable or unbearable and leaving a message to her substitute decision‐makers. In this message, she requested music from her region in Italy be played. She asked for no death notice, minimal flowers and no party. She expressed that she did not want to be a ‘vegetable’ and that she did not want her family to feel guilty for their decisions. At the end, she wrote that she would always love her family, and they would be in her heart forever.

## 4 DISCUSSION

These three vignettes illustrate the process and experiences of integrating LSW with ACP discussions and provide insight into how this can support a relational approach to autonomy during ACP. Participants were invited to contemplate their goals, values and end‐of‐life care preferences in the context of their personal history. This allowed both facilitator and participant to integrate this lived experience into their ACP discussions, supporting relational autonomy by identifying elements that may influence a person's perspectives about end‐of‐life care. For example, it was clear Mrs. Dixon's relationship with her faith and identity as a mother was significant and likely to impact her decision‐making. Rather than viewing the priest's advice as potentially an undue influence on her individual decision‐making, it was seen as a necessary element of her ability to make a choice. The guided discussions utilising LSW allowed this relationship to be integrated into her ACP.

Similarly, the LSW process facilitated discussion with family. For Mrs. Tripoli, family was involved throughout the LSW and ACP process, coming together in a relaxed social setting. End‐of‐life discussions with Italian populations are challenging due to high levels of superstition regarding talking about dying. Older Italians also tend to prefer family‐based decision‐making, which can lead them to view ACP as redundant.^30^ The ability for LSW to support family involvement, even in topics deemed taboo, may contribute to enabling ACP discussions for this community. It could also assist in adopting a family‐centric, rather than individual, approach to end‐of‐life discussions, which is preferable for many culturally and linguistically diverse populations.^31^

The process equally shows the importance of selecting substitute decision‐makers based on both their relationships with the person and shared values and beliefs. This was the case for Mrs. Dixon, who wished for only her Catholic children to be decision‐makers. The same can be seen from Mrs. Chen, whose confidence in nominating her sons as substitute decision‐makers was grounded in shared experiences of caring for her husband in his age. This is consistent with concepts of ‘relational knowing’,^32^ where substitute decision‐makers are elected based on their deep understanding of the person. For Mrs. Dixon, to know her fully is to know Catholicism, meaning only her children with this insight were suitable substitute decision‐makers. For Mrs. Chen, the shared experience of caring for her husband supported her certainty that her sons would provide the right care for her.

These stories also demonstrated the impact a relational approach can have on ACP documentation. Mrs. Chen produced a life story that included end‐of‐life wishes as well as a completed advance care directive; Mrs. Dixon wrote a letter to her children detailing her values and wishes, and Mrs. Tripoli produced a statement of values excluding end‐of‐life treatment decisions. These three formats all support a relational approach to autonomy; were Mrs. Chen to present to emergency with her end‐of‐life wishes documented, as they are, alongside her life story, it would allow her treating physicians insight into her history, which could benefit their relationship to her and the care she receives at end‐of‐life.^24^ Mrs. Dixon's letter was written with the express purpose of providing advice for family members, rather than being legally binding. Mrs. Tripoli equally wrote her directive with a view towards family‐based decision‐making rather than a document ensuring her individual autonomy, choosing to allow her family to make choices based on their relational knowing. While these formats are legally valid,^33^ it is unlikely that a person would make a document in this way with freely available resources. This approach could also support ACP facilitators presenting ACP education to incorporate a person's history and individualise approaches to ACP documentation. The integration of LSW allowed both facilitator and participant to gain greater understanding of one another and supported novel approaches to documentation that better expressed the participant's context.

### 4.1 Limitations

There are certain limitations, notably ACP documents created using this LSW process have not yet been tested in end‐of‐life situations. It is unclear how doctors will view documents, such as Mrs. Dixon's and Mrs. Tripoli's, which decline to stipulate treatment decisions. Equally, it is unclear the impact ACP of this nature will have on family members elected as substitute decision‐makers. Advance care planning documents that emphasise guiding principles not only allow flexibility for substitute decision‐makers to adapt to changing contexts but also may place substitute decision‐makers under pressure in interpreting these statements and feeling the need to make the ‘final’ decision. Further work might address this by having documents produced through the EARLI project assessed by clinicians for clinical utility across care settings.

## 5 CONCLUSIONS

Stories from the EARLI project have highlighted how LSW can assist in adopting a relational approach across the ACP continuum. The process of sharing stories can support understanding of goals and values, facilitate family involvement, promote selection of substitute decision‐makers and provide novel, person‐centred approaches to ACP documentation that can be adapted to a family‐based decision‐making approach if needed. Advanced care planning facilitators may consider integrating LSW processes into ACP discussions to support understanding of a person's social context during end‐of‐life planning. This may be useful when working with people from collectivist societies who prefer a family‐based decision‐making approach. Further research is needed to explore the impact of this approach on end‐of‐life situations, regarding both the interpretation of these documents by health‐care professionals and the impact on substitute decision‐makers.

## CONFLICT OF INTEREST STATEMENT

No conflicts of interest declared.

## Supporting information


Appendix S1

## Data Availability

Research data are not shared.

## References

[1] Karusoo‐Musumeci A , Yeoh L , Walton R , et al. Enhanced Advance Care Planning and Life Review Longitudinal Intervention (EARLI): protocol for a cluster randomized controlled cross‐over trial of life story work and facilitated advance care planning among older Australian adults in community settings. Accessed June 21, 2024. https://ssrncom/abstract=4911516 10.1016/j.cct.2024.107795 39743017

[2] Sellars M , Detering KM , Silvester W . Current advance care planning practice in the Australian community: an online survey of home care package case managers and service managers. BMC Palliat Care. 2015;14(1):15. doi:10.1186/s12904-015-0018-y 25903912 PMC4416336

[3] Detering KM , Carter RZ , Sellars MW , Lewis V , Sutton EA . Prospective comparative effectiveness cohort study comparing two models of advance care planning provision for Australian community aged care clients. BMJ Support Palliat Care. 2017;7(4):486‐494. doi:10.1136/bmjspcare-2017-001372 28918387

[4] Frechman E , Dietrich MS , Walden RL , Maxwell CA . Exploring the uptake of advance care planning in older adults: an integrative review. J Pain Symptom Manage. 2020;60(6):1208‐1222.e59. doi:10.1016/j.jpainsymman.2020.06.043 32645455 PMC7342022

[5] Silveira MJ , Kim SY , Langa KM . Advance directives and outcomes of surrogate decision making before death. N Engl J Med. 2010;362(13):1211‐1218. 20357283 10.1056/NEJMsa0907901 PMC2880881

[6] Vandervoort A , Houttekier D , Vander Stichele R , Van der Steen JT , Van den Block L . Quality of dying in nursing home residents dying with dementia: does advanced care planning matter? A nationwide postmortem study. PLoS One. 2014;9(3):e91130. 24614884 10.1371/journal.pone.0091130 PMC3948949

[7] Lewis E , Cardona‐Morrell M , Ong KY , Trankle SA , Hillman K . Evidence still insufficient that advance care documentation leads to engagement of healthcare professionals in end‐of‐life discussions: a systematic review. Palliat Med. 2016;30(9):807‐824. doi:10.1177/0269216316637239 26951066

[8] Killackey T , Peter E , Maciver J , Mohammed S . Advance care planning with chronically ill patients: a relational autonomy approach. Nurs Ethics. 2020;27(2):360‐371. doi:10.1177/0969733019848031 31122121

[9] Hickman SE , Lum HD , Walling AM , Savoy A , Sudore RL . The care planning umbrella: the evolution of advance care planning. J Am Geriatr Soc. 2023;71(7):2350‐2356. doi:10.1111/jgs.18287 36840690 PMC10958534

[10] Sudore RL , Fried TR . Redefining the “planning” in advance care planning: preparing for end‐of‐life decision making. Ann Intern Med. 2010;153(4):256‐261. doi:10.7326/0003-4819-153-4-201008170-00008 20713793 PMC2935810

[11] Fleuren N , Depla MFIA , Janssen DJA , Huisman M , Hertogh CMPM . Underlying goals of advance care planning (ACP): a qualitative analysis of the literature. BMC Palliat Care. 2020;19(1):27. doi:10.1186/s12904-020-0535-1 32143601 PMC7059342

[12] Willmott L , White B , Mathews B . Law, autonomy and advance directives. J Law Med. 2010;18(2):366‐389. 21355437

[13] Gómez‐Vírseda C , de Maeseneer Y , Gastmans C . Relational autonomy: what does it mean and how is it used in end‐of‐life care? A systematic review of argument‐based ethics literature. BMC Med Ethics. 2019;20(1):76. doi:10.1186/s12910-019-0417-3 31655573 PMC6815421

[14] Morrison RS , Meier DE , Arnold RM . What's wrong with advance care planning? JAMA. 2021;326(16):1575‐1576. doi:10.1001/jama.2021.16430 34623373 PMC9373875

[15] Chiang F‐M , Wang Y‐W , Hsieh J‐G . How acculturation influences attitudes about advance care planning and end‐of‐life care among Chinese living in Taiwan, Hong Kong, Singapore, and Australia. Healthcare. 2021;9(11):1477. 34828523 10.3390/healthcare9111477 PMC8621689

[16] Dutta O , Lall P , Patinadan PV , et al. Patient autonomy and participation in end‐of‐life decision‐making: an interpretive‐systemic focus group study on perspectives of Asian healthcare professionals. Palliat Support Care. 2020;18(4):425‐430. doi:10.1017/s1478951519000865 31699170

[17] Lin C‐P , Cheng S‐Y , Chen P‐J . Advance care planning for older people with cancer and its implications in Asia: highlighting the mental capacity and relational autonomy. Geriatrics. 2018;3(3):43. 31011081 10.3390/geriatrics3030043 PMC6319225

[18] Johnson SB , Butow PN , Kerridge I , Tattersall MHN . Patient autonomy and advance care planning: a qualitative study of oncologist and palliative care physicians' perspectives. Support Care Cancer. 2018;26(2):565‐574. doi:10.1007/s00520-017-3867-5 28849351

[19] Craig DP , Ray R , Harvey D , Shircore M . Multidisciplinary clinicians and the relational autonomy of persons with neurodegenerative disorders and an advance care plan: a thematic analysis. J Multidiscip Healthc. 2021;14:3385‐3398. doi:10.2147/jmdh.S345792 34916800 PMC8668252

[20] Willmott L , White B , Tilse C , Wilson J , Purser K . Advance health directives: competing perceptions, intentions and use by patients and doctors in Queensland. QUT Law Rev. 2013;13:30‐51.

[21] Lieblich A . Chapter 4: The introduction of life story into art therapy training programs. In: Yaguri T , Merari D , eds. Art Therapy Education: Teaching, Training, Research. Cambridge Scholars Publishing; 2021; 67.

[22] Macleod F , Storey L , Rushe T , McLaughlin K . Towards an increased understanding of reminiscence therapy for people with dementia: a narrative analysis. Dementia. 2021;20(4):1375‐1407. doi:10.1177/1471301220941275 32772555 PMC8132012

[23] McKeown J , Clarke A , Repper J . Life story work in health and social care: systematic literature review. J Adv Nurs. 2006;55(2):237‐247. doi:10.1111/j.1365-2648.2006.03897.x 16866815

[24] Nathan S , Fiore LL , Saunders S , et al. My life, my story: teaching patient centered care competencies for older adults through life story work. Gerontol Geriatr Educ. 2022;43(2):225‐238. doi:10.1080/02701960.2019.1665038 31498034

[25] Doran C , Noonan M , Doody O . Life‐story work in long‐term care facilities for older people: an integrative review. J Clin Nurs. 2019;28(7–8):1070‐1084. doi:10.1111/jocn.14718 30431682

[26] Thompson R . Using life story work to enhance care. Nurs Older People. 2011;23(8):16‐21. doi:10.7748/nop2011.10.23.8.16.c8713 22017158

[27] Thoresen L , Lillemoen L . “I just think that we should be informed” a qualitative study of family involvement in advance care planning in nursing homes. BMC Med Ethics. 2016;17(1):72. doi:10.1186/s12910-016-0156-7 27829409 PMC5103414

[28] Hilgeman M , Allen R , Snow A , Durkin D , DeCoster J . Preserving identity and planning for advance care (PIPAC): preliminary outcomes from a patient‐centered intervention for individuals with mild dementia. Aging Ment Health. 2014;18(4):411‐424. doi:10.1080/13607863.2013.868403 24359036

[29] Takenouchi S , Uneno Y , Matsumoto S , et al. Culturally adapted RN‐MD collaborative SICP‐based ACP: feasibility RCT in advanced cancer patients. J Pain Symptom Manage. 2024;68:548‐560.e2. doi:10.1016/j.jpainsymman.2024.08.037 39237027

[30] Sinclair C , Smith J , Toussaint Y , Auret K . Discussing dying in the diaspora: attitudes towards advance care planning among first generation Dutch and Italian migrants in rural Australia. Soc Sci Med. 2014;101:86‐93. doi:10.1016/j.socscimed.2013.11.032 24560228

[31] Green A , Jerzmanowska N , Green M , Lobb EA . ‘Death is difficult in any language’: a qualitative study of palliative care professionals' experiences when providing end‐of‐life care to patients from culturally and linguistically diverse backgrounds. Palliat Med. 2018;32(8):1419‐1427. doi:10.1177/0269216318776850 29767578

[32] Robins‐Browne K , Hegarty K , Guillmen M , Komesaroff P , Palmer V . The role of relational knowing in advance care planning. J Clin Ethics. 2017;28(2):122‐134. 28614075

[33] National framework for advance care planning documents. 2021.

