# Supplementary figures and images for: Could life story work support relational autonomy in advance care planning? Stories from the EARLI project
Source: Australas J Ageing. 2025 May 2;44(2):e70042. doi: 10.1111/ajag.70042 (PMC12048695; doi:10.1111/ajag.70042)

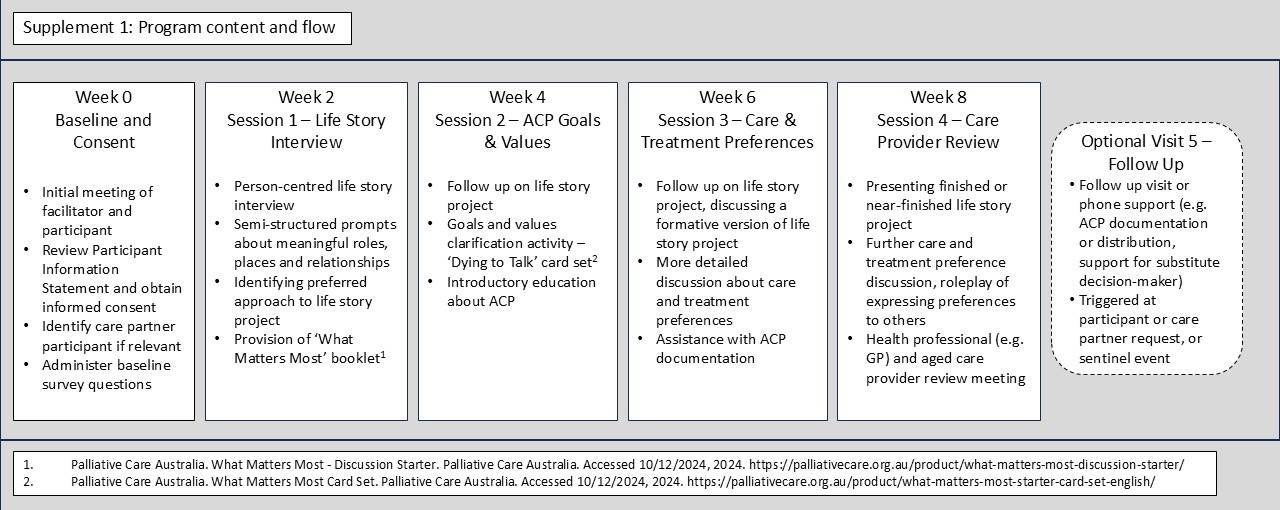

Supplement: Supplementary file 1 — Appendix S1 [file AJAG-44-0-s001.jpg]
